# The Impairing Effect of Mental Fatigue on Visual Sustained Attention under Monotonous Multi-Object Visual Attention Task in Long Durations: An Event-Related Potential Based Study

**DOI:** 10.1371/journal.pone.0163360

**Published:** 2016-09-28

**Authors:** Zizheng Guo, Ruiya Chen, Kan Zhang, Yirun Pan, Jianhui Wu

**Affiliations:** 1 School of Transportation and Logistics, Southwest Jiaotong University, Chengdu, China; 2 National United Engineering Laboratory of Integrated and Intelligent, Southwest Jiaotong University, Chengdu, China; 3 Key Laboratory of Behavioral Science, Institute of Psychology, Chinese Academy of Sciences, Beijing, China; 4 College of Psychology and Sociology, Shenzhen University, Shenzhen, China; Universita degli Studi di Roma La Sapienza, ITALY

## Abstract

The impairing effects of mental fatigue on visual sustained attention were assessed by event-related potentials (ERPs). Subjects performed a dual visual task, which includes a continuous tracking task (primary task) and a random signal detection task (secondary task), for 63 minutes nonstop in order to elicit ERPs. In this period, the data such as subjective levels of mental fatigue, behavioral performance measures, and electroencephalograms were recorded for each subject. Comparing data from the first interval (0–25 min) to that of the second, the following phenomena were observed: the subjective fatigue ratings increased with time, which indicates that performing the tasks leads to increase in mental fatigue levels; reaction times prolonged and accuracy rates decreased in the second interval, which indicates that subjects’ sustained attention decreased.; In the ERP data, the P3 amplitudes elicited by the random signals decreased, while the P3 latencies increased in the second interval. These results suggest that mental fatigue can modulate the higher-level cognitive processes, in terms of less attentional resources allocated to the random stimuli, which leads to decreased speed in information evaluating and decision making against the stimuli. These findings provide new insights into the question that how mental fatigue affects visual sustained attention and, therefore, can help to design countermeasures to prevent accidents caused by low visual sustained attention.

## Introduction

Sustained attention refers to the brain’s ability to maintain attention and remain alert to relevant stimuli appearing at unpredictable time points over long periods of time [1, 2]. These time points are random in nature. Many kinds of work, such as driving, monitoring of radar and nuclear power plant, monitoring, require operators to maintain sustained attention on multiple objects for prolonged periods. Declining in sustained attention is a critical factor that can affect task performance and operation safety [3].

One of the principal causes of decrements in sustained-attention is mental fatigue [4], which has been defined as a “state of reduced mental alertness that impairs performance” [5]. Many studies have demonstrated that the adverse effects of mental fatigue on sustained attention, reflected as decreased behavioral performance. For example, in a sustained attention to response task (SART), Bonnefond et al. [3] found that participants’ performance declined in terms of both accuracy and speed in time. In a visual task designed to examine the influence of mental fatigue on attention, Boksem et al. [6] found that as the duration of the task increase, the number of misses and false alarms increase, and the response speeds decrease. In a Go/NoGo task, Kato et al. [7] observed a significant increase in reaction times to Go stimuli, and in errors to Go and NoGo stimuli as the time spent on the task increase. In these studies, similar study patterns were adopted, i.e., participants were instructed to execute a task continuously for a long period of time, and then their behavioral performances were compared between the former and latter of the experiment.

Although behavioral performances (i.e., reaction times and accuracy rates) are the outcomes of a series of mental activities, including perception, attention, decision making, and response activation, using performance measures alone is not sufficient to identify how mental fatigue affects the process of sustained attention. Due to the high temporal resolution of Event-related potential (ERP), it’s often employed to investigate the time course of cognitive activities. The centro-parietal P3 component is the most extensively studied ERP component and is thought to reflect high-level cognitive functions during the later stages of information processing [8]. More specifically, the literature [9–11] in studies of target detection task suggests that the P3 component reflects a sign of context updating and subsequent memory storage in the evaluation of stimuli. The amplitude of the P3 component is proportional to the amount of available attentional resources [12–14] and the latency of this component reflects the speed of high-level cognitive activities [15].

Conventional investigations of mental fatigue’s influence on sustained attention, such as the SART, oddball, and Go/NoGo tasks, often use single-task paradigm, in which participants only maintain visual attention on one target at a time [3, 7, 16]. Although a few studies have adopted dual-task paradigm, they usually separate the tasks into the visual and auditory sensory channels [17]. Baddeley and Hitch [18] verified that performing two simultaneous tasks on two separate perceptual domains (i.e. a visual and a verbal task) is nearly as efficient as performing the tasks separately. In contrast, when a person tries to perform two tasks simultaneously on the same perceptual domain, performance is heavily impaired. In reality, parallel visual tasks are more common, and operators often need to visually attend to multiple objects synchronously. For example, when operating a vehicle drivers need to pay attention to surrounding vehicles to maintain safe distances and to other interrupting visual events such as traffic signals, pedestrians. To our knowledge, there is no research on the effect of mental fatigue in impairing sustained attention while participants performing visual dual-task paradigm.

Thus, the aim of this study is to explore the influence of mental fatigue on sustained attention when participants perform two tasks in the visual perceptual domain. A synchronous dual-task was employed to simulate a real-life, long-duration, multi-object, visual attention task, which included a continuous tracking-task (primary task) and a detection task (secondary task). In secondary task, stimuli appear at unpredictable intervals and positions (we call “random signal” afterwards). Using the paradigm mentioned above, the effect of mental fatigue on sustained attention was assessed by comparing the ERPs elicited by the random signals in the secondary task between the first and last intervals of the task, which had different levels of mental fatigue. We hypothesized that mental fatigue would result in a deterioration of sustained attention, reflected as a decrease in the accuracy rate of random signals in the secondary task. Furthermore, mental fatigue may impair higher-level cognitive activity associated with processing the random signals, which would be reflected as a decreased P3 amplitude and/or increased P3 latency. Apart from the P3 component, Boksem et al. (2005) reported that both the N1 and N2b amplitude modulated with time on task, which suggest that mental fatigue may also impair earlier cognitive-processing. We thus also analyzed these components.

## Methods

### Participants

Sixteen right-handed undergraduates (8 females, 8 males) that ranged in age from 18 to 24 years old (mean = 21.2, standard deviation [SD] = 1.3 years) participated in this experiment. Telephone screening prior to enrollment was conducted to exclude persons with a history of neurological or psychiatric illness and those taking specific medications that influence the central nervous system. All participants had normal or corrected-to-normal vision. Furthermore, participants were instructed to avoid alcohol drinking on the day of experiment, and to refrain from eating or exercising 2 h before the study. Written informed consent was obtained from all participants prior to the experiment. This study was approved by the Ethics Committee of Human Experimentation at Southwest Jiaotong University. The method were carried out in accordance with relevant guidelines and regulations.

### Apparatus

A 35.56-cm cathode ray tube display was used to present the experimental task, and the display was positioned 70 cm from the eyes of the participants. Participants’ responses during the tasks were made using a joystick (Logittech, F710, Switzerland). The joystick has a rocking bar for the left hand and a button for the right hand. Participants controlled the tracking circle in the primary task by rocking the bar and responded to the random signals in the secondary task by pressing the button.

Electroencephalogram (EEG) data were recorded continuously from 32 active electrodes attached to an electrode cap (Brain Products, GmbH, Munich, Germany). Electrode positions included the standard International 10–20 system locations and intermediate sites. The electrooculogram (EOG) was recorded from the outer canthi of the right eyes for HEOG and above the left eye for VEOG. FCz was used as an online reference for all channels. The EEG data from each electrode site were digitized at 1000 Hz with an amplifier band-pass filter of 0.5 to 100 Hz. The impedances of all electrodes were kept below 5kΩ.

### Experimental tasks

**Fig 1:**
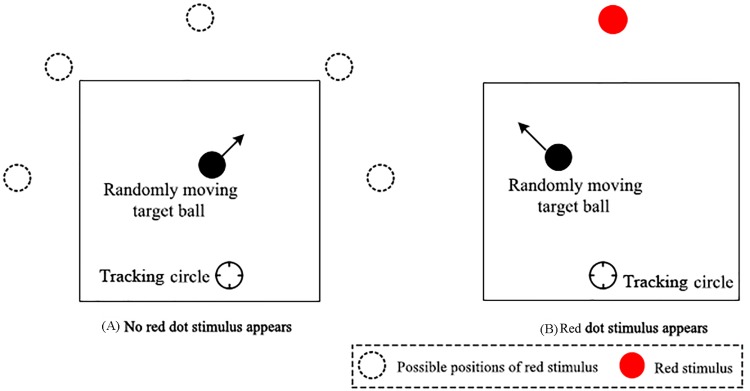
Schematic representation of the experimental task. (A) An example of the screen without the random signal (i.e., red dot). (B) An example of the screen with the random signal (i.e., red dot).

A synchronous dual-task with a continuous tracking task (primary task) and random signal detection task (secondary task) was applied in the experiment. In the continuous tracking task, a target ball (diameter = 0.8 cm) randomly moved in a square region (length of side = 10 cm), which was located at the middle of the display screen. The participants were asked to manipulate a tracking circle (inner diameter = 8 mm) with a joystick to catch the target ball and keep the center point distance between the target ball and tracking circle as small as possible. The random signal detection task was embedded in this experiment to evaluate the participants’ sustained attention. As participants performed the tracking task, a red dot would randomly appear (duration = 200 ms) at one of five possible positions, as shown in Fig 1. The visual angle of red dot is 0.66°. Once the red dot appeared, participants were instructed to respond as soon as possible. If the participants did not respond to the red dot within 1000 ms, the trial would be deemed as an omission and was invalid. During the 63-min test, the red dot appeared 243 times at random time intervals, which were within the range of 14.5 ± 2s. The participants were instructed to keep the tracking-circle aligned as closely as possible to the target ball while reacting to the red dot as soon as possible at the same time. A schematic representation of the experimental tasks is shown in Fig 1.

### Procedure

All participants were required to sleep for at least 8 h at the night before the experiment. On the day of the experiment, participants arrived at the lab at 09:00and gave us a self-report about their sleep duration which confirmed that all of them had more than 8 h sleeping duration last night, then completed a written informed consent form. Following the application of the EEG electrodes, participants were instructed to practice tasks for 5 min. Then, participants performed the normal experiment for 63 min without rest.

### Subjective sleepiness measures

Subjective sleepiness was assessed by the Karolinska Sleepiness Scale (KSS) [17, 19]. This is a 9-point scale that is scored as follows: 1 = extremely alert; 3 = alert; 5 = neither alert nor sleepy; 7 = sleepy, but no difficultly remaining awake; 9 = extremely sleepy, fighting sleep. Subjective sleepiness measurements were executed twice during the experiment. The first measurement was performed at the 26-min time point and the second was performed at the 61-min time point, and each measurement lasted 1.5 min. The participants’ KSS scores were obtained verbally by the experimenter. In addition, we calculated the times of eye blinks on the continues EEG data in two intervals to verify our experiment whether induced mental fatigue [20, 21].

### Data analysis

Three performance indexes were recorded. The first index was the center point distance between the target ball and the tracking circle (sampling rate 30 Hz) in the primary task. The other two indexes, respectively, were the participants’ reaction times and their accuracy rates to the red dot in the secondary task, which was a measure of participants’ sustained attention to the random event [1].

In order to amplify the differences of fatigue level, the data from two time intervals (the first interval: 0–25 min, the second interval: 36–60 minutes), including the behavioral measures and EEG data, were selected for analysis.

Data beyond three standard deviations were deleted. Paired-samples t-tests between the first and second intervals were performed on the subjective sleepiness scores and behavioral performance data including the mean distance between the tracking circle and the target ball, the reaction time to the random red dot and the signal detection accuracy rate to the random red dot.

For the EEG data, we only analyzed the ERPs that were time-locked to the onset of the red dots in the secondary task. We used the Brain Vision Analyzer 2.0 software (Brain Products, Munich) to process the data off-line. The EEG data were re-referenced to the mean of the left and right mastoids. Eye movements were corrected using the Gratton and Coles method [22]. A 35-Hz low-pass filter was applied to eliminate the line noise. After filtering, the EEG data were segmented into epochs, which ranged from -200 ms to 800 ms after the onset of the red dots. Epochs with artifacts, e.g., voltages exceeding ±75 μv, were rejected. The single-trial epoch was then baseline corrected and averaged to form a grand average ERP.

The N1 and N2b component which were induced by the red dot stimuli was defined as the negative peak between 170 and 240 ms, 290 and 360 ms, respectively. The data from six electrodes (C3, Cz, C4, P3, Pz and P4 for N1; F3, Fz, F4, C3, Cz and C4 for N2b) were selected for analyzing the N1 and N2b component. The P3 component was defined as the largest positive peak between 380 and 450 ms induced by the red dot stimuli. The data from six electrodes (C3, Cz, C4, P3, Pz, and P4) were selected for analyzing the P3 component. These sites were chosen in agreement with the existing literature [6, 23] and by visually inspecting the grand average ERP to determine where the N1, N2b and P3 were maximal. After measuring the peak amplitude/latency, statistical analyses using two-way repeated measures analyses of variance (ANOVAs) were performed. In the ANOVAs, the first factor was the task interval (first vs. second), and the second factor was the electrode site (F3, Fz, F4, C3, Cz and C4 for N2b; C3, Cz, C4, P3, Pz, and P4 for N1 and P3). The Greenhouse-Geisser correction method was applied for all of the repeated-measures ANOVAs that had more than one degree of freedom.

Both peak and mean amplitude measurement have advantages and disadvantages [24], thus we use both of them to confirm our result in our main target P3 component. The mean P3 amplitude was quantified as the average amplitude in the grand average wave (380–450 ms after stimulus presentation). The mean amplitude was also compared between the first and second intervals using ANOVAs.

For the KSS scores and P3 amplitude/latency, the difference values were defined as the value in the second interval minus the value in the first interval. The Pearson correlation coefficients between the KSS scores and P3 amplitude difference values and between the KSS scores and P3 latency difference values were respectively calculated at the Pz electrode, which had the maximal amplitude according to the grand average ERP results.

## Results

### Subjective sleepiness measurements

The mean KSS scores significantly increased from 2.12 (SD = 0.34) in the first interval (0–25 min) to 7.14 (SD = 0.92) in the second interval (36–60 min). The results of the paired-sample t-tests showed a significant difference in the subjective sleepiness measure (KSS scores) between the first and second intervals (t _(15)_ = -20.37, p < 0.01). Meanwhile, we found that the times of eye blinks significantly increased from 224 (SD = 69) in the first interval to 281 (SD = 86) in the second interval. The results of the paired-sample t-tests showed a significant difference in the eye closures between the first and second intervals (t _(15)_ = -4.623, p < 0.001).

### Behavioral performance

For the tracking task (primary task), pairwise comparisons between the first and second intervals indicated that the mean distance between the target ball and tracking circle increased significantly as the time spent on the task increased (t _(15)_ = -3.59, p <0 .05; 18.96 vs. 28.34 pixels).

**Fig 2:**
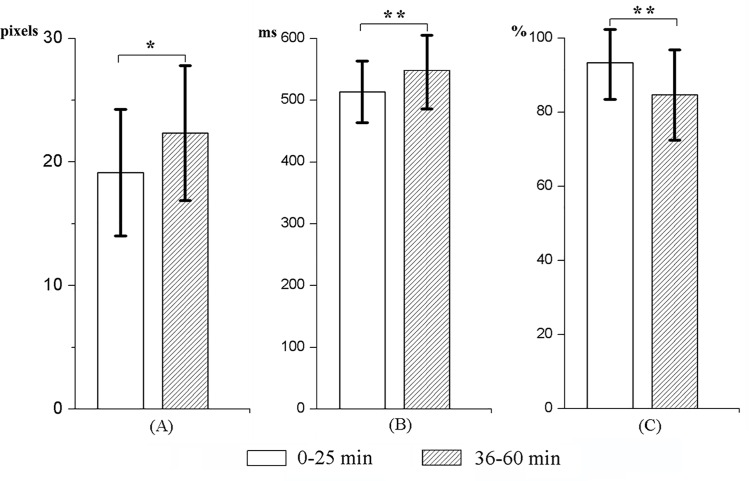
Measures of behavioral performance. (A) The mean distance between the tracking circle and the target ball. (B) The reaction time to the random red dot. (C) The signal detection accuracy rate to the random red dot. *p < 0.05, **p < 0.01.

Moreover, for the random signal detection task (secondary task), participants responded slower and the accuracy rate was lower in the second interval than in the first interval (535.69 vs. 466.56 ms and 92.40% vs. 87.35%, t _(15)_ = -4.76, p < 0.01 and t _(15)_ = -3.29, p < 0.01, respectively). The means and SDs of the behavioral measures for the two intervals are illustrated in Fig 2.

Besides, a correlation analysis between the decline in performance on the tracking task and the signal detection task was carried out. However, no significant correlation was observed between the difference value (the second interval minus the first interval) of the distance between the target ball and tracking circle and the difference value of the reaction time towards random signal (r = 0.075, p = 0.781) or the difference value of the accuracy rate towards random signal (r = 0.057, p = 0.833).

### Peak measure of P3

**Fig 3:**
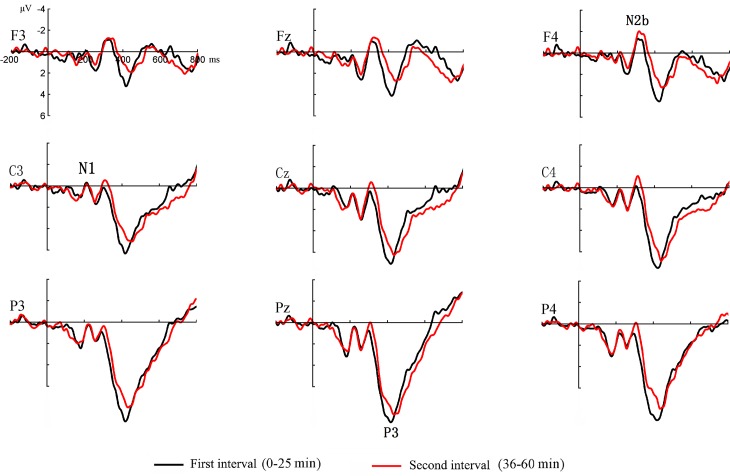
Event-related potentials elicited by the random signals (red dots) in the first and second intervals.

As shown in Fig 3, clear P3 components were elicited by the random red dots in the first and second intervals.

For the P3 amplitude, the results of the ANOVA showed significant main effects for the task interval (F _(1, 15)_ = 14.52, p < 0.01, η_p_^2^ = 0.49) and electrode site (F _(5, 75)_ = 6.06, p < 0.05, η_p_^2^ = 0.29). The P3 amplitude was more positive in the first interval, and the most positive amplitude occurred at the Pz electrode. The task interval by electrode site interaction effect was not significant (F _(5, 75)_ = 0.84, p > 0.05, η_p_^2^ = 0.05).

For the P3 latency, there was a significant main effect for the task interval (F _(1, 15)_ = 16.89, p < 0.01, η_p_^2^ = 0.53). Compared to the P3 latency in the first interval, the P3 latency in the second interval was prolonged. However, the main effect of electrode site and the task interval by electrode site interaction effect were not significant (F < 2.15, p > 0.05 for both).

### Mean P3 amplitude

**Fig 4:**
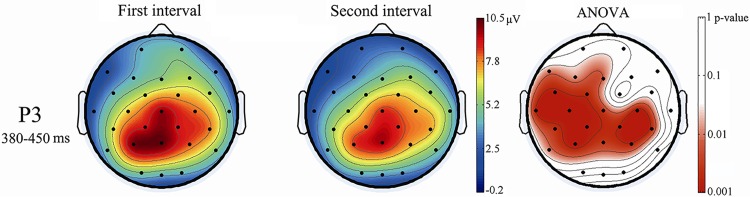
Mean event-related potential amplitudes and p-values.

Scalp topographies of the mean P3 amplitudes for the first interval (left) and the second interval (middle) were analyzed, as shown in Fig 4. The panel on the right illustrates the scalp distribution of the p-values, which was determined by performing analyses of variance statistical tests between the first and second intervals. For the mean P3 amplitude, the results of the ANOVA showed significant main effects for the task interval (F _(1, 15)_ = 6.36, p < 0.05, η_p_^2^ = 0.30) and electrode site (F _(27, 405)_ = 9.68, p < 0.01, η_p_^2^ = 0.39). Moreover, compared to the second interval, the mean P3 amplitude was more positive in the first interval at the central and parietal scalp areas. The most positive amplitude occurred at the Pz electrode. The task interval by electrode site interaction effect was not significant (F _(27, 405)_ = 0.95, p > 0.05, η_p_^2^ = 0.06).

### Correlation analyses

**Fig 5:**
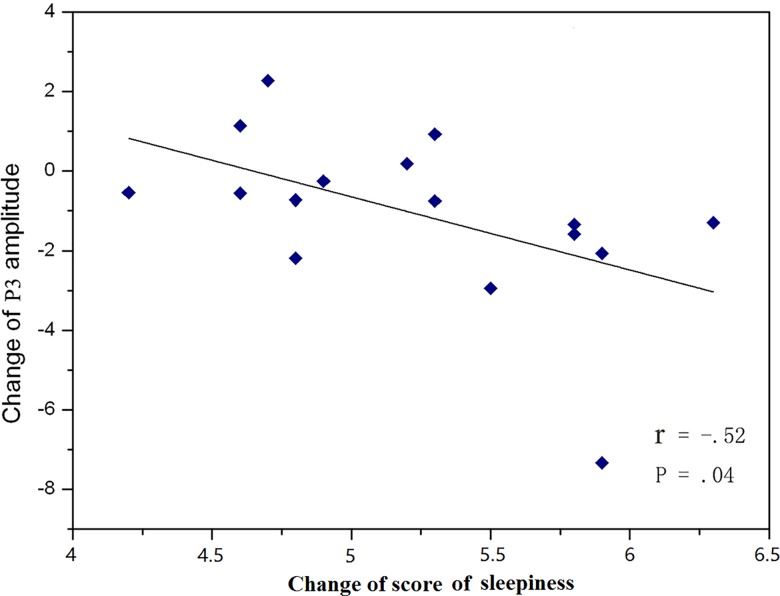
Relationship between the difference value of the subjective sleepiness scores and the P3 amplitude difference value.

The P3 amplitude difference value (the second interval minus the first interval) was significantly negatively correlated with the KSS scores difference value (r = -0.522, p = 0.043) (Fig 5). However, no significant correlation was observed between the P3 latency difference value and the KSS scores difference value (r = -0.522, p = 0.043).

### Peak measure of N1 and N2b

As shown in Fig 3, clear N1 and N2b components were elicited by the random red dots in the first and second intervals. For amplitude and latency of N1 and N2b, no significant main effect and interaction effect were found (1.22 < F < 3.63, P > 0.05).

## Discussion

The present study examined the effects of mental fatigue on sustained attention under a multi-object visual attention condition. KSS scores and the times of eye blinks significantly increased in the second interval, which indicated that the 63-min monotonous task indeed induced mental fatigue, with subjects becoming more fatigued in the second interval.

Regarding the behavioral data, compared with the first interval, the distance between the target and tracking circle in the primary task significantly increased in the second interval. Most importantly, for the random signal detection task (secondary task), the reaction times were prolonged and the accuracy rates towards the random signals decreased in the second interval compared with the reaction times and accuracy rates in the first interval. According to Parasuraman et al. [25], “sustained attention ability is mainly evaluated by the vigilance decrement, which is typified by either a decrease in the number of correct detections over time and/or an increase in [the reaction time] to signals over the watch-keeping period.” Therefore, the observed prolonged reaction times and increased omission rates towards the random signals in the second interval in the present study indicate that the sustained attention level was lower in the second interval than it was in the first interval, and imply that mental fatigue caused the decrease in sustained attention. Besides, no significant correlation was observed between the difference value of the distance between the target ball and tracking circle and the difference value of the reaction time or the difference value of accuracy rate towards random signal, which indicate that the performance on both tasks declined in parallel and that participants didn't trade off performance on one task for the other.

Furthermore, the ERP results revealed that the P3 amplitude (both the peak and mean amplitudes) in the second interval decreased significantly from that in the first interval. The correlation analysis between the P3 amplitude difference value and the KSS scores difference value further suggested that the more the participants’ fatigue increased, the more the P3 amplitude decreased. The P3 amplitude can serve as an indicator of the attentional resources available for the secondary task in a dual-task paradigm [26–28]. Here, as the mental fatigue increased, the mental resources that could be devoted to the secondary task also decreased, which was reflected as the decreased P3 amplitude. These results are consistent with the results of previous studies that used dual-task paradigms with different sensory channels, and further support the influence of mental fatigue on sustained attention during two synchronous visual tasks. In addition, in the present study, the P3 latency was significantly prolonged in the second interval. The P3 latency may reflect the speed of high-level cognitive activity, such as the stimulus-evaluation and decision-making phases [15]. Thus the increased P3 latency in the second interval might suggest that mental fatigue delayed the speed of information processing during the high-level cognitive stage [29]. This modulation of P3 latency was not observed in previous studies that used single tasks or dual tasks that crossed the visual and auditory channels. The reason for this inconformity may be that the two synchronous visual tasks occupied the same sensory channel and caused mutual interference between the primary and secondary tasks in the current study. This in turn may have increased the difficulty of the tasks, which is reflected as that the reaction time towards target detection in our experiment is obviously longer than the other single task and simultaneous tasks (crossed the visual and auditory channels) [4, 7, 16]. The increased difficulty of the red dot detection task in the current study may increase the sensitivity of the P3 component to the influence of mental fatigue.

Our results showed that mental fatigue didn't affect the amplitude or latency of N1 or N2b, this may suggest that earlier cognitive steps during detection of random signal is not affected by mental fatigue. Interestingly, Boksem et al. and Faber et al. both found that the N1 amplitude decreased with time on task, which was not observed in the current study. Boksem et al. also found the N2b amplitude increased with time on task, but Faber et al. and we didn’t. The major reasons for the differences may be due to the paradigm and the experimental design. The above two studies employed the similar paradigm (visual single-task) and stimuli (letters) that had a lot of differences compared to the current study. In addition, Boksem et al.’s experiment lasted for 3h, Faber et al.’ experiment lasted for 2h, but ours just for 63min which may not be long enough to affect the earlier visual processing.

Previous studies only investigated the influence of mental fatigue on sustained attention under single or dual-task that crossed the visual and auditory channels. However, in reality, parallel visual tasks are more common, and operators often need to visually attend to multiple objects synchronously, such as driving. Obviously, one of the novelties of the current study is that we explore the influence of mental fatigue on sustained attention under the condition of two simultaneous task that occupied the visual channel. Our results showed that mental fatigue can lead to not only less attentional resources allocated to the random stimuli, but also delayed information speed in stimulus evaluating and decisions making stage.

One limitation of the current study is that it only examined the influence of mental fatigue on visual sustained attention; whether the results from the concurrent visual tasks are valid for other types of tasks, such as parallel auditory tasks, needs to be examined in future studies. Another limitation is that subjective mental fatigue was assessed by the KSS, which is not precise enough. In the future, more precise subjective fatigue measures could be adopted, for example, the Samn-Perelli scale or the Dundee Stress State Questionnaire which were widely used in this field [30–32]. In addition, more accurate eye activity measures could be employed to measure the mental fatigue level [33].

## Conclusions

The current study demonstrated that mental fatigue induced a decrease in the participants’ sustained attention under the condition of dual synchronous visual tasks, which was reflected as prolonged reaction times and decreased accuracy rates to random signals in the secondary task. More importantly, our analyses of the ERPs elicited to random signals in the secondary task may help identify the neurological mechanisms underlying impaired visual sustained attention. Specifically, less attentional resources were allocated to the random stimuli in the secondary task, as indicated by the decreased P3 amplitude, and it took participants longer to evaluate or categorize the stimuli and context, as indicated by the delayed P3 latency during the second interval.

In summary, the findings of this study reveal the underlying neurological-mechanisms about the effect of mental fatigue on visual sustained attention. It also provided some new insights into future designs of operator-attention monitoring systems and countermeasures for the prevention of the accidents caused by decrease of sustained attention.
